# Knowledge, attitude, and perception of low back pain and activities that may prevent it among adolescents in Nigeria

**DOI:** 10.4314/ahs.v22i4.18

**Published:** 2022-12

**Authors:** Bashir Bello, Abdulbaki Aminu, Auwal Abdullahi, Mukadas O Akindele, Ushotanefe Useh, Aminu A Ibrahim

**Affiliations:** 1 Department of Physiotherapy, Faculty of Allied Health Sciences, College of Health Sciences, Bayero University, Kano, Nigeria; 2 Lifestyle Disease Research Entity, Faculty of Health, Northwest University, Mafikeng Campus, South Africa; 3 Department of Physiotherapy, Muhammad Abdullahi Wase Teaching Hospital, Hospitals Management Board, Kano, Kano State, Nigeria

**Keywords:** Low back pain, prevention, adolescents, knowledge, attitude, perception

## Abstract

**Background:**

Awareness of activities that may result in low back pain (LBP) among adolescents is fundamental in preventing adulthood LBP.

**Objective:**

The aim of this study was to investigate adolescents' knowledge, attitude, and perception of LBP and activities that may prevent LBP in Kano, North-western, Nigeria.

**Methods:**

This was a cross-sectional survey involving 400 school-going adolescents recruited using a multistage random sampling technique. Data was analysed using descriptive statistics and Chi-square test with 0.05 set as level of significance.

**Results:**

The mean age of the respondents was 16.0±1.50 years. LBP annual prevalence was 34.2%, with more girls (31.1%) reporting having LBP compared to boys (28.4%). More than half (59.3%) of the respondents had poor knowledge of LBP and activities that may prevent it. However, they had a good attitude (63%) and perception (74%) of LBP and activities that may prevent it. There was no significant association of levels of knowledge, attitude, and perception of LBP and activities that may prevent LBP with gender, age, and class of study (p > 0.05).

**Conclusion:**

Adolescents in Kano, North-western Nigeria had poor knowledge of LBP and activities that may prevent it. Therefore, there is a need to embark on an LBP prevention program among adolescents in Kano, North-western Nigeria.

## Background

Low back pain (LBP) is a growing and serious public health concern in children and adolescents, especially among the school-age population^1^. It is believed that a first episode of LBP during adolescence mainly results in LBP in adulthood 2. LBP is said to be a disease of the old and not young and as such adolescent LBP has received less attention. Previous researchers^2^–^4^ have stated that adolescents' LBP is of great concern because of its impact on their spine and insufficient physical activity, absenteeism from school, and risk of developing severe LBP in the future. Pellisé et al.^5^ affirmed that LBP in adolescents had been considered unusual and a harbinger of serious organic diseases until some epidemiologic data dispelled the misconception that it is unique to adults only. LBP can become recurrent, especially over 15 years, suggesting that chronic LBP in adults could commence in adolescence 4 and continue into adulthood.

Low back pain could be ubiquitous among adolescents due to their participation in many activities without good strength in the abdominal musculature and the spine extensors. With limited flexibility of the hamstring muscles^6^,^7^. According to Pellisé et al.^5^, about 39.8% of adolescents suffer from LBP, limiting daily activities in almost 10–40% affected. Nigeria has an estimated 200 million people with 22.3% of adolescents^8^. Previous reports have shown that LBP mainly emerges in the early teen years. Almost one-third of 14-year-olds report pain in the lower back, and approximately 10% report a very persistent LBP2^9^. A study conducted in the South Western part of Nigeria reported a lifetime, twelve-month, one month and point prevalence rates of adolescent low back pain as 58.0%, 43.8%, 25.6%, and 14.7%, respectively in secondary school students aged 10–19 years in Ibadan^10^. Another study by Akinpelu et al.^11^ reported LBP prevalence among adolescents in a Nigerian community (Ibadan) with a 12-month prevalence of 41.7% boys 39.8% for girls. Bello and Toriola^12^ also reported a prevalence of adolescent LBP as 56.3% among secondary school students in Kano, North-western Nigeria. Thus, LBP among adolescents is increasingly becoming a public health problem that warrants more attention than previously thought. Several risk factors for adolescent LBP have been mentioned, including age, gender, obesity, decreased flexibility and mobility of muscles, hypermobility, sports, type and way of lifting weight, poor posture, physical activity level, smoking, and domestic habits such as watching TV and computer/videogame as well as carrying heavy backpacks^12^–^14^. Modifying these factors may help prevent LBP among adolescents.

Public awareness and education on knowledge of the rapid physical, mental, and social changes during adolescence helps individuals to absorb and adapt to these changes, preventing them from becoming victims of many serious illnesses such as LBP^15^. Attitude and perceptions can affect the behaviour and recovery of a patient as well. According to Silcock et al.^16^, negative attitudes and perceptions regarding LBP increase the severity and extent of disability caused by the disorder. Previous history and onset of LBP among adolescents can be associated with back problems in adulthood, implying that prevention in adolescence may positively impact maturity^17^. Hestbaek et al.^4^ affirmed that adolescents must be targeted to change their attitudes and promote good health for a primary intervention to become a reality. Coenen et al.^3^ argued that knowledge of LBP among adolescents is one area that has limited researches and needs to be explored to identify how best to prevent and manage the problem among this vulnerable population. Literature is still scarce on adolescents' knowledge, attitude, and perceptions towards LBP prevention in Nigeria. Hence, this study aimed to determine adolescents' knowledge, attitude, and perception of LBP and activities that may prevent it in Kano, North-western Nigeria.

## Methods

### Study design

This study was a cross-sectional survey involving four hundred adolescents across Kano State secondary schools, recruited using a multi-stage sampling technique.

### Participants

The populations for the study were adolescents from various registered private and public secondary schools in Kano, North-western Nigeria. Participants were included in the study if they 1 were male or female aged 14–19 years from any of the three senatorial zones of Kano State, 2 had experienced LBP defined as pain or discomfort felt between the 12th ribs and inferior gluteal folds the buttock area. They were excluded if they are adolescents with any physical deformity, known causes of back pain, had undergone low back surgery, and had LBP related to menstrual periods.

### Questionnaire

A self-developed questionnaire with close-ended questions was used to collect information regarding the participants' demographic variables, knowledge, attitude, and perception of LBP and activities that may prevent it. The questionnaire consists of four sections. The first section was about demographic information and experience (prevalence) of LBP. The second section consists of 20 questions about the knowledge level of adolescents on LBP and activities that may prevent it. Each question has a possible score of 0 or 1 hence the lowest score is 0 and the highest score is 20. The third section consists of 15 questions regarding the attitude level of adolescents on LBP and activities that may prevent it. The fourth section consists of 15 questions about the perception of adolescents on LBP and activities that may prevent it. Each question in the third and fourth sections are rated using a Likert scale ranging from 0 (strongly agree) to 4 (strongly disagree). The questionnaire was tested for face and content validity, and the results showed that it was easy to comprehend and had good content validity.

### Procedure

A multistage random sampling technique was used to recruit 400 school-going adolescents from twelve wards in Kano Municipal Council, Kano State, Nigeria. First, six wards were selected from twelve wards using a simple random technique (balloting method). Second, a simple random technique was used to select one school each from the six wards resulting in total of six schools. Third, a stratified random sampling technique was used to select the three classes (SS 1, SS 2, and SS 3) from each school, with different classes taken as the strata. Finally, systematic sampling technique was used to select adolescent from each class to complete the total number of adolescents required. After ensuring their eligibility through screening, written consent of the participants was obtained. The designed questionnaire was then self-administered. They were then informed to ask for assistance if they encounter any problems with the questionnaire items.

### Data analysis

The data was obtained, described, and summarized using descriptive statistics of mean, standard deviation, frequencies, and percentages. Chi-square was used to assess the association of levels of knowledge, attitude, and perception of LBP and activities that may prevent it with certain perceived correlates. These correlates were demographic factors such as gender, age, and class of study. All statistical analysis was performed at 0.05 alpha levels using Statistical Package for Social Sciences (SPSS) version 20.0 software (SPSS Inc. Chicago, Illinois, USA).

## Results

### Characteristics of the study population

Of the 400 respondents, 183 (45.8%) were boys and 217 (54.2%) were girls. The mean age of the respondents was 16.02±1.50 years (range 14–19 years). The prevalence of LBP was 34.2%, with a boy and girl prevalence of 28.4% and 39.1%, respectively (Table 1). Demographic characteristics of the respondents are fully shown in Table 1.

**Table 1:** Demographic characteristics of the respondents (n = 400)

Variable	
Age (years), mean±SD	16.02±1.50
14 years, *n* (%)	74 (18.5)
15 years, *n* (%)	100 (25)
16 years, *n* (%)	66 (16.5)
17 years, *n* (%)	87 (21.8)
18 years, *n* (%)	47 (11.8)
19 years, *n* (%)	26 (6.5)
Gender, n (%)	
Boy	183 (45.8)
Girl	217 (54.2)
Class of study, *n* (%)	
SSS1	187 (46.8)
SSS2	88 (22)
SSS3	125(31.3)
School type, *n* (%)	
Public	200 (50.0)
Private	200 50.0)
Prevalence of low back pain, *n* (%)	
Overall	137 (34.2)
Boys	52 (28.4)
Girls	85 (39.1)

SD, standard deviation; SS, secondary school; SSS, senior secondary school

### Levels of knowledge, attitude, and perception of LBP and activities that may prevent it among the respondents

The mean knowledge, attitude, and perception scores were 8.56±3.04, 51.7±9.88, 57.8±11.4, respectively as shown in Table 2. The majority of the respondents (59.3%) had poor knowledge of LBP and perceived activities that may prevent it. However, the results indicated that most respondents had a good attitude (63%) and perception (74%) of LBP and activities that may prevent it (Table 2).

**Table 2:** Levels of knowledge, attitude, and perception of LBP and activities that may prevent it among the respondents

Variable	
**General knowledge**, mean±SD	8.56±3.04
Category, *n (%)*	26 (6.5)
Very poor	26 (6.5)
Poor	237 (59.3)
Good	117 (29.3)
Very good	20 (5.0)
**General attitude**, mean±SD	51.7±9.88
Category, *n (%)*	
Very poor	5 (1.2)
Poor	5 (1.2)
Good	143 (35.8)
Very good	252 (63.0)
**General perception**, mean±SD	57.8±11.4
Category, *n (%)*	
Very poor	0 (0.0)
Poor	80 (20)
Good	296 (74)
Very good	24 (6.0)

SD, standard deviation

### Association of levels of knowledge, attitude, and perception of LBP and activities that may prevent it with selected demographic variables

As shown in Table 3, Chi-square statistics revealed no significant association (p > 0.05) between the levels of knowledge, attitude, and perception of LBP and activities that may prevent it with gender, age, and class of study (Table 3).

**Table 3:** Associations of levels of knowledge, attitude, and perception of LBP activities that may prevent it with selected demographic variables

	Variable	Chi-square	*df*	*p*-value
Knowledge	Gender	5.33	3	0.149
	Age	33.84	15	0.728
	Class of study	14.27	6	0.243
Attitude	Gender	0.6	2	0.74
	Age	15.2	10	0.12
	Class of study	7.74	4	0.10
Perception	Gender	2.07	2	0.35
	Age	8.42	10	0.58
	Class of study	3.45	4	0.48

## Discussion

This study aimed to evaluate adolescents' knowledge, attitude, and perception of LBP and activities that may prevent it in Kano, North-western Nigeria. In this study, the annual prevalence of LBP among adolescents in the Kano metropolis was 34.2%. This result aligns with several national and international studies^12^,^18^, which reported LBP prevalence in adolescents to vary between 13.7% and 60.3%. In addition, various physiological, genetics and physical factors with time watching television (TV), or using the computer and carrying backpacks and demographics and socioeconomic status, have all been associated with LBP among adolescents^13^. However, in this study, most participants who experienced LBP, when asked, mentioned having pain either at the lower back, waist or buttocks and attributed it to factors such as carrying heavy backpacks, prolonged sitting, and sitting posture.

Regarding gender, results from this study are as per previous findings^19^–^21^, which showed that girls had the highest prevalence of LBP, with 20.7% boys and 50.6% girls, respectively. However, de Vitta et al.^18^ found LBP prevalence in 35.6 % of boys and 64.4% of girls in their study, indicating adolescent females are more likely to present with LBP, possibly due to menarche and differences in pain perception threshold between genders. Furthermore, girls are said to enter their growth phase before boys, and this finding may reflect a direct relationship between the rapid growth of musculoskeletal structures and LBP^22^. Finally, Diepenant et al.^23^ opined that girls are likely to recognize and accept their pain symptoms associated with LBP than boys naturally due to their masculinity.

### Knowledge of LBP and activities that may prevent it

The health of adolescents and, even more importantly, their knowledge, attitude, and practices are regarded as essential factors in predicting the epidemiological transition of a population^24^. Therefore, knowledge of LBP among adolescents is fundamental to prevent them from having back problems in the future. This study's participants are within the ages of 14 to 19 years which is in line with WHO definition of adolescence^24^ and the age range of participants in previous studies on adolescent LBP 5,25. However, most of the participants in this study had a low score regarding their knowledge of LBP and activities that may prevent it, which is a cause for concern. Thus, awareness programs to educate and enlighten adolescents of LBP and activities to avoid it become necessary.

Previous studies determined the level of knowledge of LBP and physical activity/exercise in adolescents^26^,^27^ no significant associations among knowledge, gender, and age of participants with LBP. These findings are aligned with the present results, which showed no significant association between knowledge of LBP and activities that may prevent it and gender. In this study, no significant association was recorded between knowledge of LBP and activities that may prevent it and age^28^. Foltran et al. 28 showed that knowledge of LBP and activities that may prevent adolescents from it might increase with age. However, this was contrary to the present finding, which showed no association between increased knowledge of LBP and age among the adolescents and predicted that this knowledge is not likely to change without any conscious effort to raise awareness and educate the adolescents. On the other hand,

### Attitude towards activities that may prevent LBP

Identifying adolescents' attitudes and perceptions regarding the prevention of LBP is necessary because they go hand in hand with preventing it later in life. Negative attitudes and beliefs on pain have been confirmed to be a barrier to achieving the desired treatment outcomes^29^. Thus, a positive attitude and perceptions are essential in preventing LBP 30.

The majority of the present study's participants had a good score regarding attitude, which is in line with a previous study of^31^, which stated over 70% of adolescents had a good attitude to LBP prevention. Although, in this study, about 37% of the adolescents had a poor level of attitude towards activities that may prevent LBP, which still indicates a cause for concern to enhance the health of these adolescents.

No significant association between the attitude of the participants and gender was reported. Although, females have a low pain threshold and are likely to report pain and seek health care than men^32^,^33^. Anecdotal evidence shows that females take better care and pay more attention to their bodies than males. Thus, gender did not affect the level of attitudes of the participants in this study.

Houben et al.^34^ stated that a good attitude could decrease the functional disability of persons with LBP. Also, less positive beliefs and pain attitudes are associated with the persistence of pain and high levels of low back pain-related disability. Furthermore, Silcock et al.^16^ observed that a good attitude would decrease LBP-related functional disability. Therefore, a possible reason for reduced occurrence of LBP among subjects with good mood is that attitude reflects one's behaviour, which turns to habits that are carried on into later life.

Although this study showed a very good level of attitudes among adolescents towards LBP, there are areas in which their perspectives are not appropriate. As knowledge can influence a person's mood, increasing adolescents' understanding of LBP can further improve their attitude towards LBP.

### Perception of LBP and activities that may prevent it

Knowledge is critical to changing attitudes and perceptions. Patients' understanding of LBP is vital, as this knowledge promotes compliance and alters bad attitudes and perceptions, thereby improving an individual's overall quality of life^35^.

Female participants had higher perceptions scores compared to their male counterparts. However, no significant association was found between the level of perception of the participants and gender in this study. Thus, even though females take better care of their bodies and handle it with care, unlike males, gender did not affect the perception level of the participants. Attitude and perception go hand in hand, meaning a positive attitude will result in a positive perception. Furinghetti et al.^36^ alluded that perception is a condition for knowledge, and there is only a very slim line between perception and knowledge. Perceptions are considered subjective knowledge of an individual because perception is based on personal experiences and understanding^36^. In this view, it could be seen that knowledge, attitude, and perception are interrelated, and they could influence each other in one way or another. Also, the finding that no significant association was found between the perceptions level and age of the participants indicates that their perception had no relationship with age as it does not increase with age.

## Strength and limitation of the study

The strength of this study lies in the ability to analyse the understanding of adolescents on LBP and perceived factors that may prevent it. Thus, this study has provided a means of preventing LBP at an early age and as they go into adulthood. However, a significant limitation of the study is difficulty identifying the most common word that could be understood as LBP among adolescents. Moreover, being a cross-sectional study, cause and effect interpretation of the findings is not possible.

## Implications for practice and research

This present study showed that the knowledge of LBP and factors that may prevent it among adolescents in Nigeria is low. Therefore, we believe this is an essential message for musculoskeletal clinicians and adolescent health practitioners and researchers to pay attention to the need for a substantial effort to increase awareness of LBP and prevent it among adolescents in Nigeria.

## Conclusion

This study concluded that most adolescents in Kano, North-western Nigeria had poor knowledge of LBP and activities that may prevent it. However, they had a good attitude and perception of LBP and activities towards its prevention. Therefore, there is a need to embark on an LBP prevention program among adolescents in Kano, North-western Nigeria.

## Ethical considerations

This study was approved by the research ethics committee of College of Health Sciences, Bayero University, Kano, Nigeria (Ref: BUK/CHS/HREC/140). Approval of the various school authorities involved in the study was obtained. Also, informed consent was obtained from all the study participants before being recruited into the study.
